# IGF2BP3/CTCF Axis–Dependent NT5DC2 Promotes M2 Macrophage Polarization to Enhance the Malignant Progression of Lung Squamous Cell Carcinomas

**DOI:** 10.1111/crj.70031

**Published:** 2024-11-06

**Authors:** Jifeng Sun, Hao Wang, Ran Zhang, Xiaoxuan Sun, Zhanbo Wu, Jun Wang, Yuwen Wang

**Affiliations:** ^1^ Department of Radiotherapy Tianjin Cancer Hospital Airport Hospital Tianjin China; ^2^ Department of Breast Cancer Tianjin Cancer Hospital Airport Hospital Tianjin China; ^3^ Department of Thoracic Oncology Tianjin Cancer Hospital Airport Hospital Tianjin China; ^4^ Cancer Immunotherapy Department Tianjin Cancer Hospital Airport Hospital Tianjin China; ^5^ Department of Radiation Oncology Tianjin Medical University Cancer Institute and Hospital, National Clinical Research Center for Cancer, Tianjin's Clinical Research Center for Cancer, Key Laboratory of Cancer Prevention and Therapy Tianjin China

**Keywords:** CTCF, IGF2BP3, lung squamous cell carcinoma, NT5DC2

## Abstract

**Background:**

Lung squamous cell carcinoma (LUSC) is a type of lung cancer that develops in the squamous cells. It is known to be promoted by the activation of various signaling pathways and the dysregulation of key regulatory molecules. One such molecule, 5′‐nucleotidase domain containing 2 (NT5DC2), has been identified as a critical regulator in various cancers including lung cancer. However, there are no data regarding its role in LUSC.

**Methods:**

The mRNA expression of insulin‐like growth factor 2 mRNA–binding protein 3 (IGF2BP3), CCCTC‐binding factor (CTCF), and NT5DC2 was analyzed using quantitative real‐time polymerase chain reaction (qRT‐PCR), whereas their protein expression was assessed using a western blotting assay. Cell proliferation was determined using a cell counting kit‐8 (CCK‐8) assay. Cell apoptosis, CD11b expression, and CD206 expression were analyzed using flow cytometry. Tube formation was assessed through a tube formation assay. Glucose consumption, lactate production, and ATP levels were measured using colorimetric methods. The effect of NT5DC2 on the malignant progression of LUSC cells was analyzed using a xenograft mouse model assay. The levels of transforming growth factor‐beta 1 (TGF‐β1) and interleukin‐10 (IL‐10) were detected using enzyme‐linked immunosorbent assays. The associations among IGF2BP3, CTCF and NT5DC2 were identified using dual‐luciferase reporter assay, RNA immunoprecipitation assay and m6A RNA immunoprecipitation assay.

**Results:**

The expression of NT5DC2 was found to be upregulated in LUSC tissues and cells when compared with normal lung tissues and normal human bronchial epithelial cells. Silencing of NT5DC2 inhibited LUSC cell proliferation, tube formation, glycolysis, M2 macrophage polarization, and tumor formation while inducing cell apoptosis. In addition, CTCF was found to transcriptionally activate NT5DC2 in LUSC cells. IGF2BP3 stabilized the mRNA expression of CTCF through m6A methylation. Further, overexpression of CTCF or NT5DC2 attenuated the effects of IGF2BP3 silencing in both NCI‐520 and SK‐MES‐1 cells.

**Conclusion:**

The IGF2BP3/CTCF axis–dependent NT5DC2 promotes M2 macrophage polarization, thereby enhancing the malignant progression of LUSC. This study was the first to reveal the role of NT5DC2 in LUSC and the underlying mechanism. The result suggests that targeting the IGF2BP3/CTCF/NT5DC2 axis may have clinical significance in the treatment of LUSC.

## Graphical abstract

IGF2BP3 increased CTCF expression to promote the transcriptional process of NT5DC2, thus accelerating M2 macrophage polarization, which in turn increased cell proliferation, tube formation, and glucose metabolism and inhibited cell apoptosis, ultimately promoting the malignant progression of LUSC.

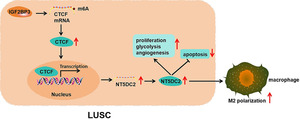

## 1 Introduction

Non–small cell lung cancer (NSCLC) accounts for the predominant subset of lung cancer cases [1]. Lung squamous cell carcinoma (LUSC) accounts for a significant proportion of NSCLC cases and is often diagnosed at a late stage when the prognosis is poor [2]. Targeted therapy holds significant promise as a cutting‐edge approach in the treatment landscape of lung cancer [3, 4]. However, there is lacking in the treatment of LUSC using targeted therapy methods. This may be attributed to a multitude of factors, including the substantial tumor heterogeneity, the intricate nature of the underlying pathophysiological mechanisms, and the limitations of animal models in accurately replicating the human tumor microenvironment [5, 6].

Tumor‐associated macrophages (TAMs), commonly called M2‐polarized macrophages, are a specific subset of macrophages that are prevalent within the tumor microenvironment [7]. These cells are believed to play a significant role in tumor development by promoting angiogenesis, facilitating tumor cell invasion and metastasis, and suppressing antitumor immune responses [8, 9, 10]. Recent research has shown that altering the signaling pathways and metabolic characteristics of TAMs can support their protumorigenic functions. For example, the activation of specific signaling pathways in TAMs produces protumor cytokines and chemokines, which promote tumor growth and metastasis [11]. Additionally, changes in the metabolic profile of TAMs can lead to the production of tumor‐promoting metabolites, which in turn can foster the growth and survival of tumor cells [12]. Elucidating the signaling and metabolic pathways that govern the functions of TAMs within the tumor microenvironment is a critical area of investigation that holds significant promise for the advancement of cancer treatment strategies.

5′‐Nucleotidase domain containing 2 (NT5DC2) belongs to the NT5DC family. This family of proteins is involved in the hydrolysis of 5′‐nucleotides, which are important for various cellular processes, including DNA and RNA metabolism [13]. NT5DC2 contains a haloacid dehalogenase (HAD) motif, a conserved sequence found in proteins that are members of the HAD superfamily. The HAD superfamily is a large and diverse group of enzymes that catalyze the dehalogenation of haloacids and play important roles in cancer progression by enhancing tumor cell growth and migration [14, 15]. Studies have shown that NT5DC2 is upregulated in several types of cancers, such as hepatocellular carcinoma [16] and NSCLC [17]. In particular, NT5DC2 exhibits the capacity to drive the M2 polarization of macrophages in breast cancer [18] and TAM recruitments in colorectal carcinoma [19]. However, the exact mechanisms by which NT5DC2 regulates tumor development of LUSC, particularly with regard to macrophage polarization, are not fully understood.

RNA‐binding proteins can specifically recognize and bind to RNA molecules to regulate various aspects of RNA metabolism. Insulin‐like growth factor 2 mRNA–binding protein 3 (IGF2BP3) is one of the most studied RNA‐binding proteins known for its specific binding to m6A‐modified RNA [20]. It is associated with various diseases such as cancers and cardiovascular diseases [21, 22]. In particular, IGF2BP3 contributed to lung cancer progression [23]. CCCTC‐binding factor (CTCF) is a highly conserved nuclear protein that can organize chromatin into topologically associated domains (TADs) and the regulation of gene expression [24]. TADs are compact, looped structures within the chromatin fiber that allow for spatial proximity of distal genomic regions, facilitating transcriptional regulation and other nuclear processes [25]. CTCF is also a transcriptional regulator that can directly bind to DNA and influence the transcription of target genes [26]. In lung cancer progression, it has been reported that CTCF promoted tumor progression [27]. Based on the above contents, the study was designed to analyze the role of NT5DC2 in the malignant progression of LUSC and macrophage polarization and the underlying mechanism.

## 2 Materials and Methods

### 2.1 Clinical Samples

In this project, a retrospective collection was made of data from 36 patients with LUSC who underwent surgical treatment at Tianjin Cancer Hospital Airport Hospital. The transportation processes for the adjacent tissues and cancerous tissues involved the use of dry ice. Sample numbers and collection dates were recorded on the outer wall of the cryovials using a marker pen. Appropriate‐sized tissue blocks were cut on a laminar flow bench and stored at −80°C. All tumor tissues were confirmed as LUSC through pathological testing. The clinical characteristics of patients with LUSC are shown in Table S1. This study was approved by the Ethics Committee of Tianjin Cancer Hospital Airport Hospital and was signed by the participants.

### 2.2 Cell Culture

The cells used in the study including normal human bronchial epithelial cells (BEAS‐2B, Procell, Wuhan, China), LUSC cells (SK‐MES‐1, Procell), human umbilical vein endothelial cells (HUVECs, EK‐Bioscience, Shanghai, China), and human embryonic kidney cells (293T, EK‐Bioscience) were cultured in DMEM (Procell). The other cells including LUSC cells (NCI‐H520, EK‐Bioscience) and human acute monocytic leukemia cells (THP‐1, EK‐Bioscience) were maintained in RPMI‐1640 medium (Procell). An eight‐character rocking method was gently used to shake the culture dish to ensure uniform distribution of cells throughout the dish. The dish was then placed in a 37°C, 5% CO_2_ incubator for cultivation. The growth of the cells was observed daily, and the nutrient solution was replaced every 24–48 h. When the cell confluence reached 70%–90%, subculturing was performed with a splitting ratio ranging from 1:2 to 1:3 to maintain cellular viability and optimal growth conditions.

### 2.3 Cell Transfection

Small interfering RNAs of NT5DC2 (sh‐NT5DC2), CTCF (sh‐CTCF), IGF2BP3 (sh‐IGF2BP3), the overexpression plasmids of NT5DC2 (oe‐NT5DC2) and CTCF (oe‐CTCF), and controls were provided by GenePharma (Shanghai, China). Passing through subculture, cells with a growth density of 80%–90% were cultivated in 10 cm diameter cell culture dishes. Once cell density reached 70%–80%, culture medium was replaced with fresh complete DMEM (Procell) or RPMI‐1640 (Procell). At the same time, cells were prepared for transient transfection of siRNAs/lipofectamine 2000 (Invitrogen, Carlsbad, CA, USA) and plasmids/lipofectamine 2000 mixture. The mixtures were added to the culture wells and cultured at 37°C.

### 2.4 Quantitative Real‐Time Polymerase Chain Reaction (qRT‐PCR)

The lysis of lung tissues and cultured cell samples was carried out using Trizol (Invitrogen). The extracted RNAs were quantified by a spectrophotometer. High‐purity RNA samples were selected, and cDNA was synthesized using a reverse transcription kit (Invitrogen). qRT‐PCR analysis was performed in different groups of cells and tumor tissues using SYBR Green reagent (TaKaRa, Dalian, China), with the 2^−ΔΔCT^ method applied for normalization and calculation of mRNA levels. Primer sequences are shown in Table 1.

**TABLE 1:** Primer sequences used for qRT‐PCR.

Name		Primer sequences (5′‐3′)
IGF2BP3	Forward	TTCAAGGACGCCAAGATCCC
	Reverse	TATCCAGCACCTCCCACTGT
CTCF	Forward	ATTGAACCTGAGCCAGAGCC
	Reverse	AGCTGTTGGCTGGTTCTGTT
β‐actin	Forward	CTTCGCGGGCGACGAT
	Reverse	CCACATAGGAATCCTTCTGACC
NT5DC2	Forward	TGGCTGCCTGGATGAAAGAG
	Reverse	GTAGGTGGGGTTGTGGAAGG
CD206	Forward	GCCTCGTTGTTTTGCGTCTT
	Reverse	GAGAACAGCACCCGGAATGA
IL‐10	Forward	ACACATCAGGGGCTTGCTC
	Reverse	GAAGTGGGTGCAGCTGTTCT
TGF‐β1	Forward	GGAAATTGAGGGCTTTCGCC
	Reverse	CCGGTAGTGAACCCGTTGAT

### 2.5 Western Blotting Assay

An appropriate amount of lysis buffer (Beyotime, Shanghai, China) was added to cell and tissue samples. Following this, the samples were lysed on ice for 10 min. The samples were then disrupted using an ultrasonic dismembrator. Protein samples were mixed with loading buffer (Beyotime) and heated at 100°C for 10 min to denature proteins, after which the protein samples and protein marker were loaded into SDS‐PAGE gels (Beyotime) for protein band separation. After electrophoresis, the proteins were incubated sequentially with primary and secondary antibodies including anti‐NT5DC2 (1:1000, Thermo Fisher, Waltham, MA, USA), anti‐VEGF‐A (1:1000, Affinity, Nanjing, China), anti‐GLUT1 (1:1000, Affinity), anti‐CTCF (1:1000, Affinity), anti‐IGF2BP3 (1:1000, Affinity), anti‐β‐actin (1:8000, Affinity), and Goat‐anti‐Rabbit IgG (1:5000, Affinity). The processed membranes were then placed flat on a scanner for imaging after staining using eyoECL Plus (Beyotime).

### 2.6 Cell Counting Kit‐8 (CCK‐8)

After transfection, cell suspension concentration was measured and adjusted, followed by seeding into each well of 96‐well plates. Further culture was performed in the incubator for 3 days. On days 1, 2, and 3, CCK‐8 solution (Beyotime) was added, and the optical density was measured using a microplate reader.

### 2.7 Flow Cytometry

After 48 h of transfection, LUSC cells were digested with trypsin, collected by centrifugation, and resuspended in 1× Annexin V‐FITC binding buffer (Solarbio, Beijing, China). Annexin V‐FITC (Solarbio) was added in the dark for 15 min. Propidium iodide (PI, Solarbio) was added before flow cytometry analysis.

### 2.8 Tube Formation Assay

Matrigel (Abwbio, Shanghai, China) was evenly spread in 48‐well plates for 30 min. Twenty‐four hours post‐transfection, the supernatant was collected and stored at −80°C. HUVECs were prepared as cell suspensions. After the Matrigel solidified, 200 μL of cell suspension was added and incubated for 18 h in a cell culture incubator. The number of inner tube formations was observed under a microscope.

### 2.9 Glucose Consumption Analysis

The experiment utilized a Glucose Detection Kit (Applygen, Beijing, China). The cells were transfected and then cultured for 48 h. The samples were diluted appropriately, and standard substances, test samples, and working solutions were added to the detection wells. The prepared reaction system was placed in a 37°C water bath for 20‐min incubation. The wavelength for absorbance detection on a microplate reader was set to 490 nm, and the optical density value of each well was measured.

### 2.10 Lactate Production

LUSC cells were transfected and cultured for 48 h. The culture medium was sampled and reserved for testing. The samples were diluted appropriately and mixed with a standard substance (Jiancheng Bioengineering Institute, Nanjing, China) and a color developer (Jiancheng Bioengineering Institute) in the detection wells. The mixture was then thoroughly mixed and then placed in a water bath to react. Samples were analyzed using a microplate reader.

### 2.11 ATP Level Analysis

ATP levels were analyzed using a commercial ATP detection kit (Beyotime). In brief, LUSC cells with various transfections were lysed using lysis buffer (Beyotime), and cell supernatants were collected by centrifugation. Working solution was added to each sample and incubated for 4 min. The samples and standard substance were added to the detection wells, followed by analysis using a luminometer.

### 2.12 Xenograft Mouse Model Assay

Male BALB/c nude mice (6 weeks old, Hunan Slyke Jingda Experimental Animal Co., LTD, Changsha, China) were used for the study. The skin was disinfected with 75% alcohol, and NCI‐H520 cell suspension (1 × 10^6^ cells) was injected subcutaneously into the right shoulder of the nude mice. The tumor growth was observed every week after inoculation for 7 days. The mice were sacrificed with pentobarbital sodium (40 mg/kg, Lianshuo Biological Technology Co, Ltd., Shanghai, China) after 5 weeks, and the subcutaneous tumors were removed, measured for size and weight, and photographed. The animal experiments were ratified by the Animal Ethical Committee of Tianjin Cancer Hospital Airport Hospital.

### 2.13 Immunohistochemistry (IHC) Assay

The embedded paraffin sections were placed on a heating block for dewaxing. The tissue sections were subjected to microwave irradiation and incubated with an appropriate amount of endogenous peroxidase inhibitor (Phygene, Fuzhou, China) for 10 min. Then, 3% BSA (Phygene) was added to cover the tissue sections evenly and incubated. The sections were quickly treated with primary solutions of Ki67 (1:1000, Affinity) and NT5DC2 (1:30, Thermo Fisher) and secondary antibody solution (Phygene). DAB (Phygene) was used for staining, followed by hematoxylin counterstaining for 4–5 s. Finally, the sections were examined under a microscope.

### 2.14 Macrophage Polarization Assay

THP‐1 cells were seeded into the lower chamber of Transwell inserts (Costar, Shanghai, China) with a pore size of 0.4 μm for culture. When cell density reached approximately 80%, the cells were induced to differentiate into macrophages with PMA (Genetimes Technology, Shanghai, China) for 24 h; after which, PMA was removed by washing with PBS. Subsequently, transfected LUSC cells were seeded on the top of the Transwell for noncontact co‐culture with the PMA‐induced differentiated THP‐1 cells. After 48 h, the differentiated macrophages from the bottom of the Transwell were harvested, and the positive percent of CD11b and CD206 was detected by flow cytometry using anti‐CD11b (Elabscience, Wuhan, China) and anti‐CD206 (Elabscience) antibodies. The mRNA expression of CD206, IL‐10, and TGF‐β1 in the cells was analyzed using qRT‐PCR. The levels of TGF‐β1 and IL‐10 in the supernatant of the bottom cells were analyzed using enzyme‐linked immunosorbent assays (ELISAs) kits, including the Human TGF‐β1 ELISA Kit (PT880, Beyotime) and the Human IL‐10 ELISA Kit (PI528, Beyotime).

### 2.15 Dual‐Luciferase Reporter Assay

Well‐growing LUSC cells were seeded into 96‐well plates. Wild‐type and mutant reporter plasmids (NT5DC2‐WT and NT5DC2‐MUT) were co‐transfected into the cells with CTCF shRNA and sh‐NC using lipofectamine 2000 (Invitrogen). Firefly Luciferase Assay Buffer (Solarbio) was added to each well of cells and incubated for 10 min. The relative luciferase activity was measured by a luminometer. Then, Renilla Luciferase Assay Buffer (Solarbio) was added, followed by measurement using a luminometer.

### 2.16 RNA Immunoprecipitation (RIP)

LUSC cells transfected with CTCF shRNA and sh‐NC were lysed with lysis buffer (Beyotime), and the cell pellet was collected by centrifugation and placed on ice. Similarly, untreated LUSC cells were processed using the same method. The antibodies against CTCF (1:30, Abcam), IgG (1:100, Abcam), and IGF2BP3 (1:100, Abcam) were added to Protein A/G beads (Millipore, Bradford, MA, USA) and incubated for 3 h with rotation. The supernatant was discarded after centrifugation. RIP Immunoprecipitation Buffer (Millipore) was added to the Beads‐Antibody mixture, and protein lysis buffer was added to the mixture containing the beads. After washing with lysis Buffer, RNA was extracted and the enrichment of NT5DC2 and CTCF was verified by qRT‐PCR.

### 2.17 m6A RNA Immunoprecipitation Assay (MeRIP)

According to the introduction of a MeRIP m6A kit (Millipore), LUSC cells transfected with sh‐IGF2BP3 or sh‐NC were collected and fragmented. IP Buffer was used to dissolve and wash the Magna Chip protein A/G beads. Subsequently, the beads were incubated with an m6A antibody and normal IgG antibody. The MeRIP reaction mixture was then added to the magnetic bead antibody mixture and incubated for 2 h. After separation with a magnetic rack, the supernatant was removed. The supernatant was purified, and CTCF expression was analyzed using qRT‐PCR technology.

### 2.18 Analysis of CTCF mRNA Stability

After transfecting IGF2BP3 and sh‐NC into LUSC cells for 24 h, the cells were treated with actinomycin D (1 μg/mL, Abcam) for 0, 2, 4, and 6 h. Total RNA was extracted, and qRT‐PCR was performed to detect CTCF expression.

### 2.19 Statistical Analysis

Data were analyzed statistically using GraphPad Prism 8.0. Differences between groups were analyzed using Student's *t* test or one‐way ANOVA. Results are presented as mean ± standard deviation. A *P* value <0.05 was considered statistically significant for indicating differences between groups.

## 3 Results

### 3.1 NT5DC2 Expression Was Upregulated in LUSC Tissues and Cells

**FIGURE 1:**
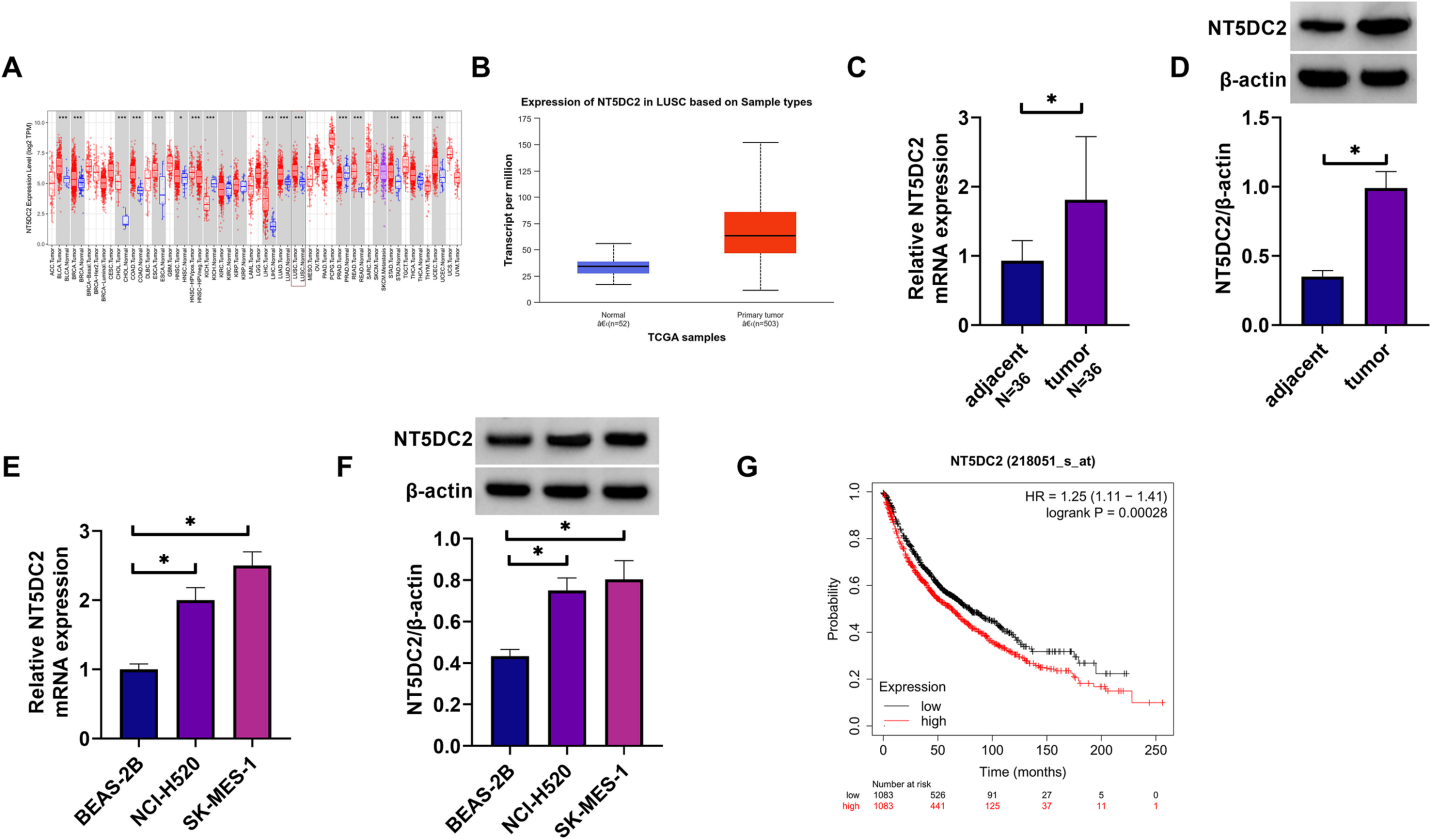
NT5DC2 expression was upregulated in LUSC tissues and cells. (A) The TIMER database is used to visualize the differential expression of NT5DC2 in the Pan‐Cancer dataset of TCGA. (B) NT5DC2 expression analysis through the TCGA database. (C) NT5DC2 mRNA expression was analyzed by qRT‐PCR in LUSC tissues and paracancerous lung tissues. (D) NT5DC2 protein expression was assessed by western blotting assay in LUSC tissues and paracancerous lung tissues. (E) NT5DC2 mRNA expression was analyzed by qRT‐PCR in BEAD‐2B cells, NCI‐H520 cells and SK‐MES‐1 cells. (F) NT5DC2 protein expression was assessed by western blotting assay in BEAD‐2B cells, NCI‐H520 cells and SK‐MES‐1 cells. (G) The Kaplan–Merier plotter database was performed to analyze the prognosis of LUSC patients with high or low NT5DC2 expression. *P < 0.05.

The TIMER database is employed to visualize the differential expression levels of NT5DC2 across the Pan‐Cancer dataset derived from The Cancer Genome Atlas (TCGA). The results showed that its expression was upregulated in various types of cancer tissues including LUSC tissues when compared with normal tissues (Figure 1A). Its high expression in LUSC tissues, as analyzed through the TCGA database, was shown in Figure 1B. Subsequently, the analysis of clinical LUSC tissues and lung tissues showed that NT5DC2 expression at mRNA and protein levels was upregulated in cancer tissues (Figure 1C,D). Moreover, the mRNA and protein expression of NT5DC2 was upregulated in LUSC cell lines including NCI‐520 and SK‐MES‐1 when compared with the levels in BEAS‐2B cells (Figure 1E,F). The result also showed that LUSC patients with high NT5DC2 expression showed a low survival rate compared with those with low NT5DC2 expression (Figure 1G). Thus, these data demonstrate that NT5DC2 is highly expressed in LUSC tissues and cells.

### 3.2 NT5DC2 Silencing Inhibited the Malignant Growth of LUSC Cells

**FIGURE 2:**
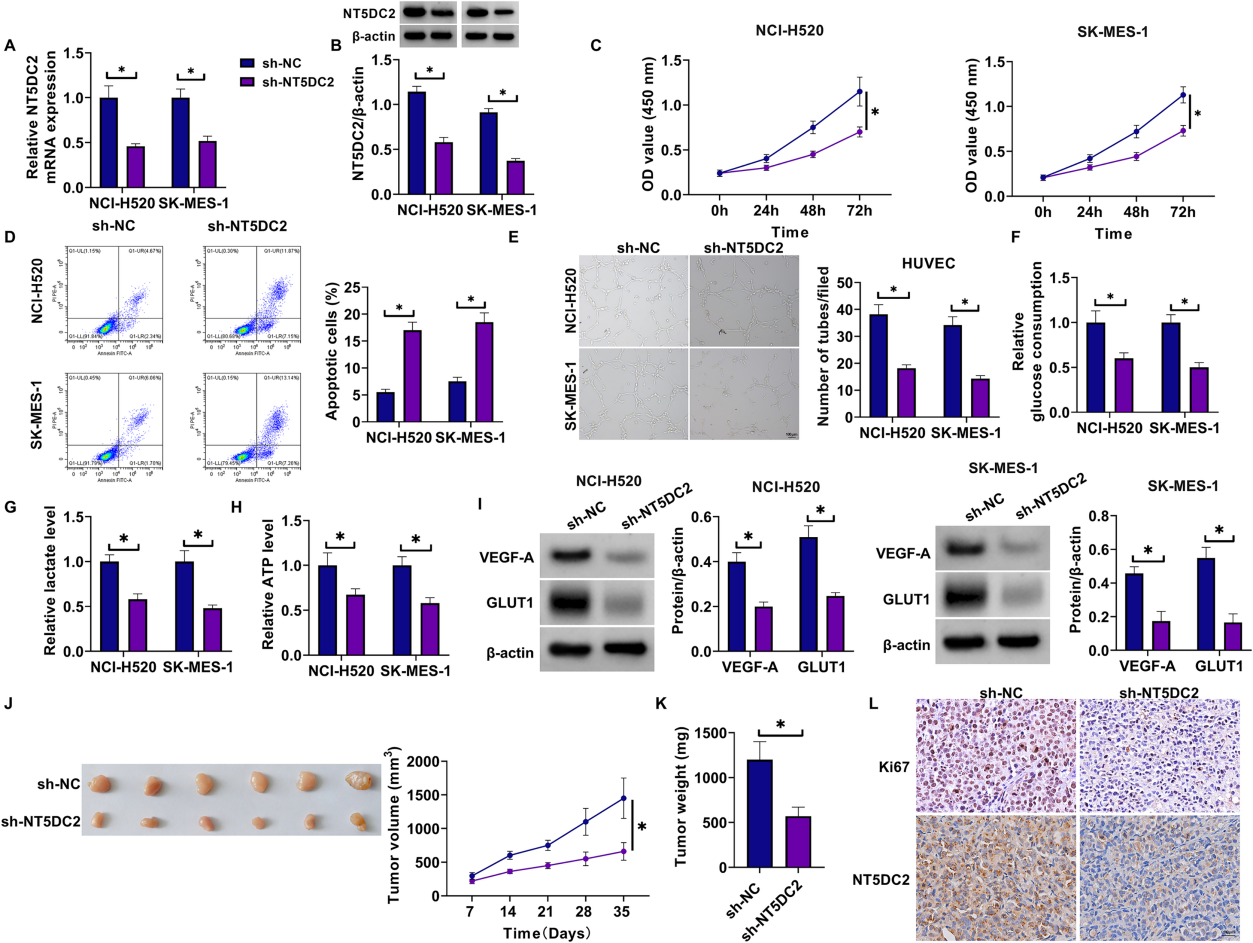
NT5DC2 silencing inhibited LUSC cell proliferation, tube formation, glycolysis and tumor formation and induced cell apoptosis. NCI‐520 and SK‐MES‐1 cells were transfected with sh‐NT5DC2 or sh‐NC. (A) NT5DC2 mRNA expression was analyzed by qRT‐PCR. (B) NT5DC2 protein expression was assessed by western blotting assay. (C) CCK‐8 assay was conducted to determine cell proliferation. (D) Flow cytometry was applied to analyze cell apoptosis. (E) Tube formation was analyzed through the tube formation assay. (F,G) Colorimetric methods were used to analyze glucose consumption, lactate production and ATP levels. (I) VEGF‐A and GLUT1 protein expression were analyzed through the western blotting assay. (J–L) NCI‐H520 cells expressing sh‐NT5DC2 or sh‐NC were injected into nude mice, and the resulting tumors were harvested after 35 days for tumor volume and tumor weight analysis (J,K), as well as for the analysis of Ki67 and NT5DC2 protein expression (L). *P < 0.05.

**FIGURE 3:**
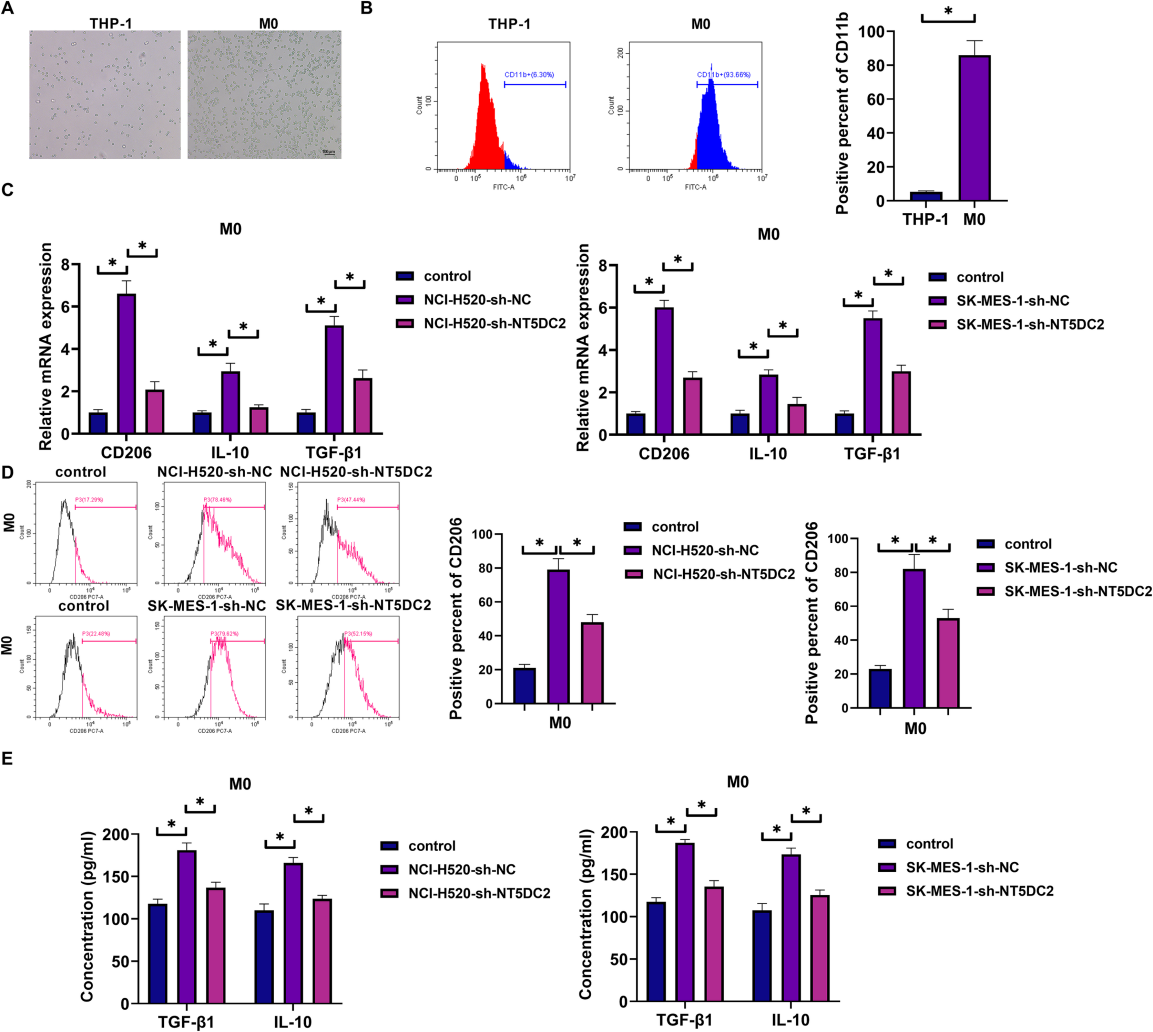
NT5DC2 silencing inhibited M2 macrophage polarization. THP‐1 cells were cultured in the bottom layer of transwell plates and induced with PMA for 24 h to differentiate into macrophages. After washing with PBS to remove PMA, the NCI‐520 and SK‐MES‐1 cells transfected with sh‐NT5DC2 or sh‐NC were inoculated into the upper layer of the transwell plates and co‐cultured with THP‐1 cells induced by PMA for 48 h. (A) Analysis of cellular morphology of THP‐1 cells and M0 cells. (B) Flow cytometry was applied to detect CD11b expression. (C) The mRNA expression of CD206, IL‐10 and TGF‐β1 was analyzed by qRT‐PCR. (D) Flow cytometry was applied to detect CD206 expression. (E) ELISAs were performed to detect the levels of TGF‐β1 and IL‐10. *P < 0.05.

The study then analyzed the effect of NT5DC2 silencing on the malignant progression of LUSC cells (NCI‐H520 and SK‐MES‐1) by transfecting the shRNA targeting NT5DC2 and sh‐NC. The result showed that NT5DC2 expression at mRNA and protein levels was significantly downregulated in both NCI‐H520 and SK‐MES‐1 cells after transfection with sh‐NT5DC2 (Figure 2A,B). Subsequently, the results showed that NT5DC2 knockdown inhibited cell proliferation and induced cell apoptosis (Figure 2C,D). The study also revealed that NT5DC2 silencing inhibited tube formation and decreased glucose consumption, lactate production, and ATP levels (Figure 2E–H). Vascular endothelial growth factor A (VEGF‐A) is secreted by endothelial cells and contributes to the homeostasis of blood vessels [28]. Glucose transporter 1 (GLUT1) is responsible for glucose uptake and is overexpressed in solid cancers [29]. The present work revealed that NT5DC2‐deficient cells showed decreases in VEGF‐A and GLUT1 protein expression (Figure 2I). The in vivo data also revealed their inhibitory effects on tumor growth and the positive expression rates of Ki67 and NT5DC2 in tumors resulting from NCI‐H520 (Figure 2J–L). THP‐1 cells were cultured in the bottom layer of transwell plates and induced with PMA for 24 h to differentiate into macrophages. The cellular morphology of THP‐1 cells and M0 cells is shown in Figure 3A. THP‐1 cells are typically round or oval in shape. PMA‐induced THP‐1 cells are larger than THP‐1 cells and have a more elongated shape. The flow cytometry revealed that CD11b expression was higher in PMA‐induced THP‐1 cells than in THP‐1 cells (Figure 3B). These results demonstrate that PMA‐induced THP‐1 cells to differentiate into macrophages. After washing with PBS to remove PMA, the NCI‐520 and SK‐MES‐1 cells transfected with sh‐NT5DC2 or sh‐NC were inoculated into the upper layer of the transwell plates and co‐cultured with THP‐1 cells induced by PMA for 48 h, followed by the analysis of CD206, IL‐10 and TGF‐β1. As shown in Figure 3C–E, the treatment with NT5DC2‐deficient cells inhibited their production. Thus, NT5DC2 silencing inhibits LUSC cell proliferation, tube formation, glycolysis, tumor formation, and M2 macrophage polarization and induced cell apoptosis.

### 3.3 CTCF Transcriptionally Activated NT5DC2 in LUSC Cells

**FIGURE 4:**
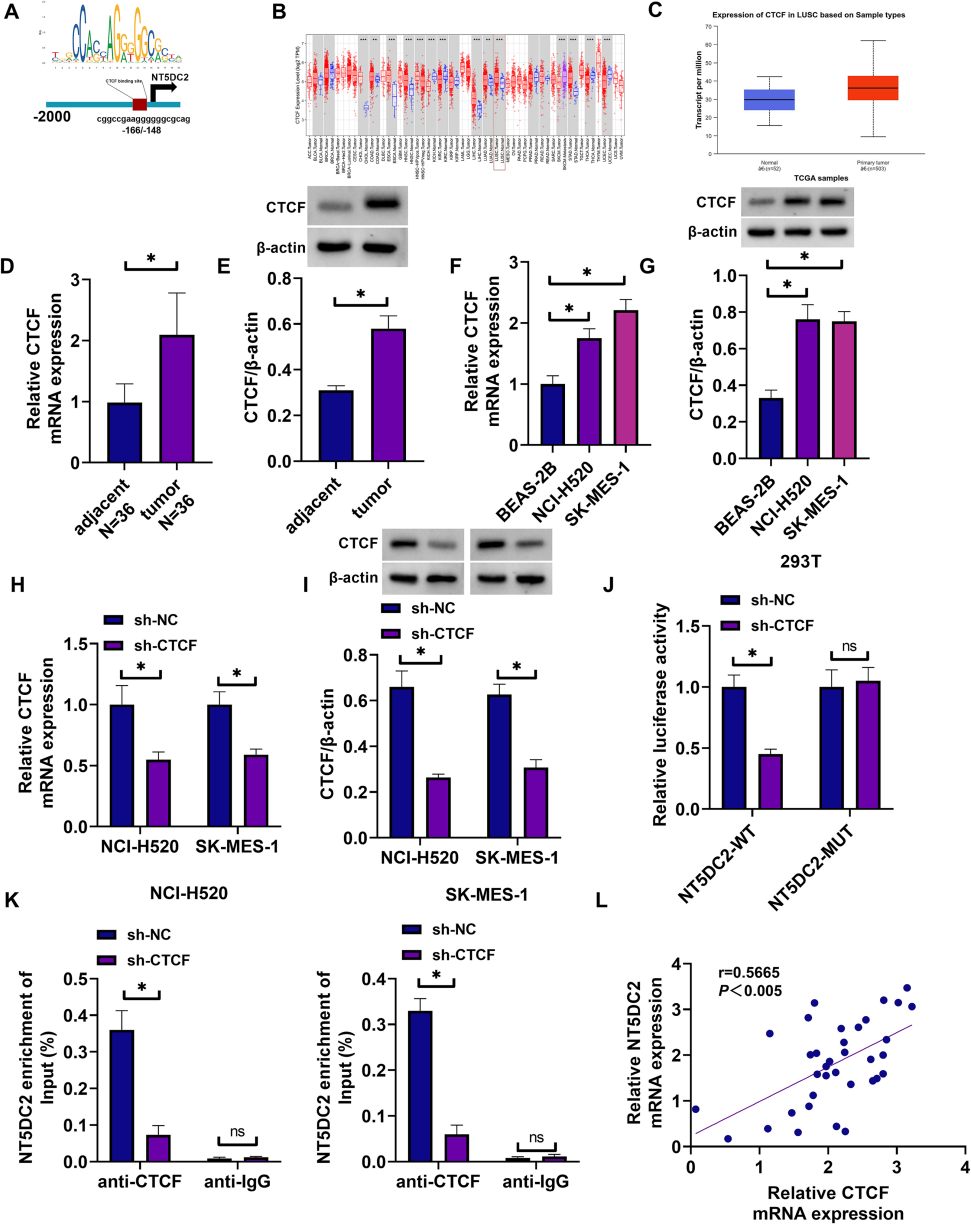
CTCF transcriptionally activated NT5DC2 in LUSC cells. (A) The schematic diagram showed the binding sites of CTCF for the promoter region of NT5DC2. (B) The TIMER database is used to assess the differential expression of CTCF in the Pan‐Cancer dataset of TCGA. (C) CTCF expression analysis through the UALCAN database. (D) CTCF mRNA expression was analyzed by qRT‐PCR in LUSC tissues and paracancerous lung tissues. (E) CTCF protein expression was assessed by western blotting assay in LUSC tissues and paracancerous lung tissues. (F) CTCF mRNA expression was analyzed by qRT‐PCR in BEAD‐2B cells, NCI‐H520 cells and SK‐MES‐1 cells. (G) CTCF protein expression was assessed by western blotting assay in BEAD‐2B cells, NCI‐H520 cells and SK‐MES‐1 cells. (H,I) The mRNA and protein expression of CTCF was analyzed in both NCI‐520 and SK‐MES‐1 cells transfected with sh‐CTCF or sh‐NC. (J,K) Dual‐luciferase reporter assay and RIP assay were performed to identify the association between CTCF and NT5DC2 in both NCI‐520 and SK‐MES‐1 cells. (L) Spearman correlation analysis was performed for NT5DC2 and CTCF mRNA expression in LUSC tissues. *P < 0.05. ns: not significant.

The schematic diagram showed that the promoter region of NT5DC2 contained the binding sites of CTCF (Figure 4A), indicating the potential regulation of CTCF in the transcriptional process of NT5DC2. As shown in Figure 4B, CTCF expression was upregulated in various types of cancer tissues including LUSC tissues in comparison with normal tissues, as analyzed through the TIMER database. Its high expression in LUSC tissues, as analyzed through the UALCAN database, is shown in Figure 4C. Subsequently, CTCF expression at mRNA and protein levels was upregulated in cancer tissues (Figure 4D,E). The mRNA and protein expression of CTCF was upregulated in both NCI‐520 and SK‐MES‐1 cells (Figure 4F,G). The study transfected CTCF shRNA and control (sh‐NC) with NT5DC2 reporter plasmids (NT5DC2‐WT and NT5DC2‐MUT) into 293T cells to identify the association between CTCF and NT5DC2. The efficiency of CTCF shRNA in downregulating CTCF expression was high and the result is shown in Figure 4H,I. As presented in Figure 4J, CTCF silencing significantly inhibited the luciferase activity of wild‐type NT5DC2 reporter plasmid but not that of mutant reporter plasmid. Moreover, the affinity of CTCF protein to NT5DC2 mRNA was high in both NCI‐520 and SK‐MES‐1 cells (Figure 4K). Further, NT5DC2 mRNA expression showed a positive correlation with CTCF mRNA expression in LUSC tissues (Figure 4L). Thus, CTCF functioned as a transcriptional regulator of NT5DC2 in LUSC cells.

### 3.4 IGF2BP3 Stabilized mRNA Expression of CTCF Through m6A Methylation

**FIGURE 5:**
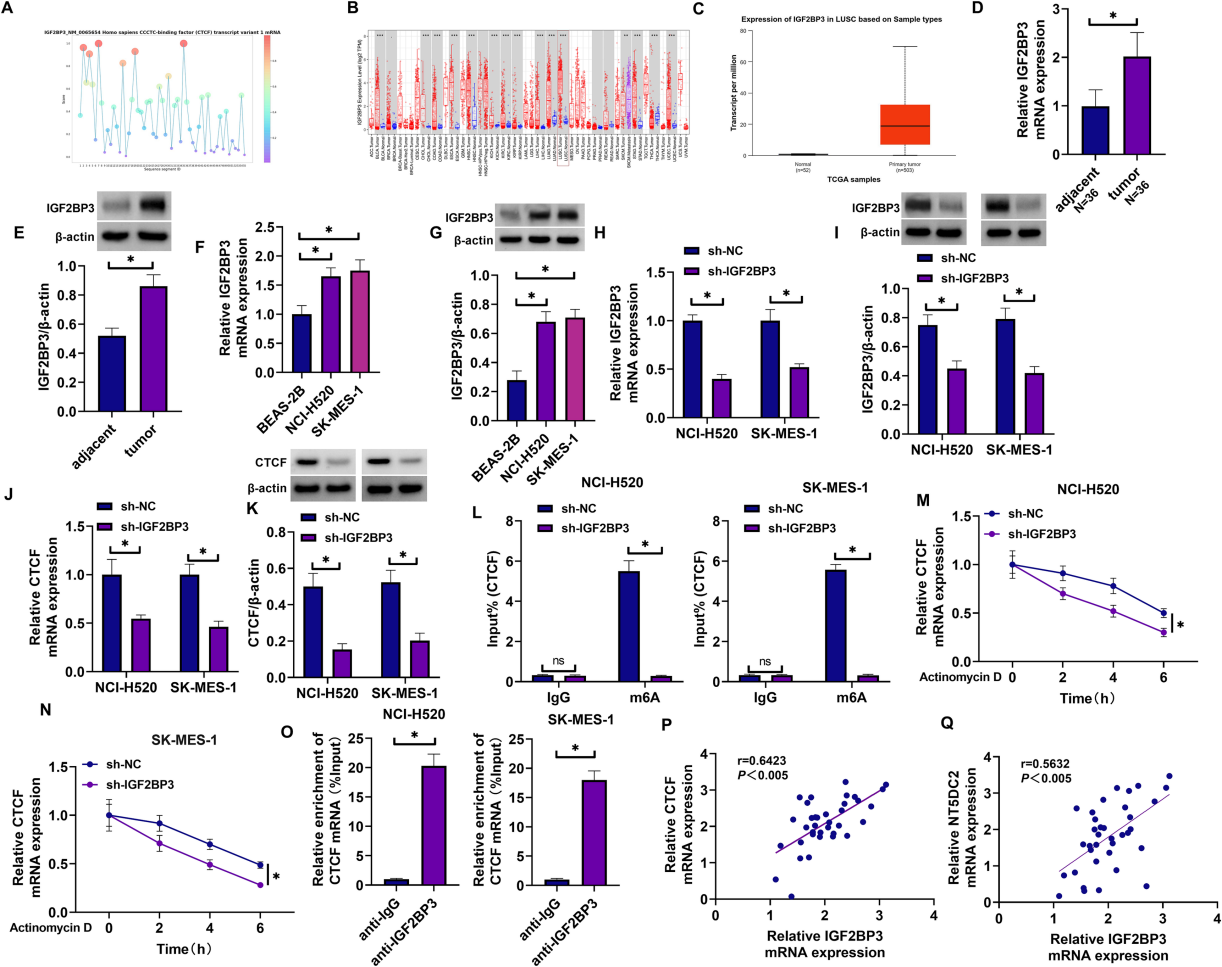
IGF2BP3 stabilized mRNA expression of CTCF through m6A methylation. (A) The binding sites of IGF2BP3 for CTCF were predicted using the RBPsuite database. (B) The TIMER database is used to assess the differential expression of IGF2BP3 in the Pan‐Cancer dataset of TCGA. (C) IGF2BP3 expression analysis through the UALCAN database. (D) IGF2BP3 mRNA expression was analyzed by qRT‐PCR in LUSC tissues and paracancerous lung tissues. (E) IGF2BP3 protein expression was assessed by western blotting assay in LUSC tissues and paracancerous lung tissues. (F) IGF2BP3 mRNA expression was analyzed by qRT‐PCR in BEAD‐2B cells, NCI‐H520 cells and SK‐MES‐1 cells. (G) IGF2BP3 protein expression was assessed by western blotting assay in BEAD‐2B cells, NCI‐H520 cells and SK‐MES‐1 cells. (H,I) IGF2BP3 expression analysis in both NCI‐520 and SK‐MES‐1 cells transfected with sh‐IGF2BP3 or sh‐NC. (J,K) The effect of IGF2BP3 silencing on CTCF mRNA and protein expression in both NCI‐520 and SK‐MES‐1 cells. (L) The MeRIP assay was performed to analyze the effect of IGF2BP3 silencing on the m6A methylation of CTCF. (M,N) The effect of IGF2BP3 depletion on the transcript half‐life of CTCF was analyzed through the actinomycin D assay. (O) The Co‐IP assay was performed to identify the association of IGF2BP3 and CTCF. (P,Q) The correlation between IGF2BP3 and CTCF or NT5DC2 in LUSC tissues was determined through the Spearman correlation analysis. *P < 0.05. ns: not significant.

The subsequent prediction showed that CTCF contained the binding sites of IGF2BP3 (Figure 5A). IGF2BP3 expression was upregulated in various types of cancer tissues including LUSC tissues in comparison with normal tissues, as analyzed through the TIMER database (Figure 5B). Its high expression in LUSC tissues, as analyzed through the UALCAN database, is shown in Figure 5C. Subsequently, IGF2BP3 expression at mRNA and protein levels was upregulated in LUSC tissues compared with normal lung tissues (Figure 5D,E). The mRNA and protein expression of IGF2BP3 was upregulated in both NCI‐520 and SK‐MES‐1 cells (Figure 5F,G). The high efficiency of IGF2BP3 shRNA in downregulating IGF2BP3 expression was confirmed by qRT‐PCR and western blotting assays (Figure 5H,I). Subsequent data revealed that IGF2BP3 silencing inhibited the mRNA and protein expression of CTCF in both NCI‐520 and SK‐MES‐1 cells (Figure 5J,K). The MeRIP assay showed that the affinity of m6A protein to CTCF mRNA was decreased after IGF2BP3 silencing (Figure 5L). As shown in Figure 5M,N, IGF2BP3 silencing shortened the transcript half‐life of CTCF mRNA. Moreover, the affinity of IGF2BP3 protein to CTCF mRNA was high in both NCI‐520 and SK‐MES‐1 cells (Figure 5O). Further, IGF2BP3 expression was positively correlated with CTCF expression and NT5DC2 expression in LUSC tissues (Figure 5P,Q). Thus, IGF2BP3 mediated the m6A methylation process of CTCF in LUSC cells.

### 3.5 Overexpression of CTCF or NT5DC2 Attenuated IGF2BP3 Silencing–Induced Effects in Both NCI‐520 and SK‐MES‐1 Cells

**FIGURE 6:**
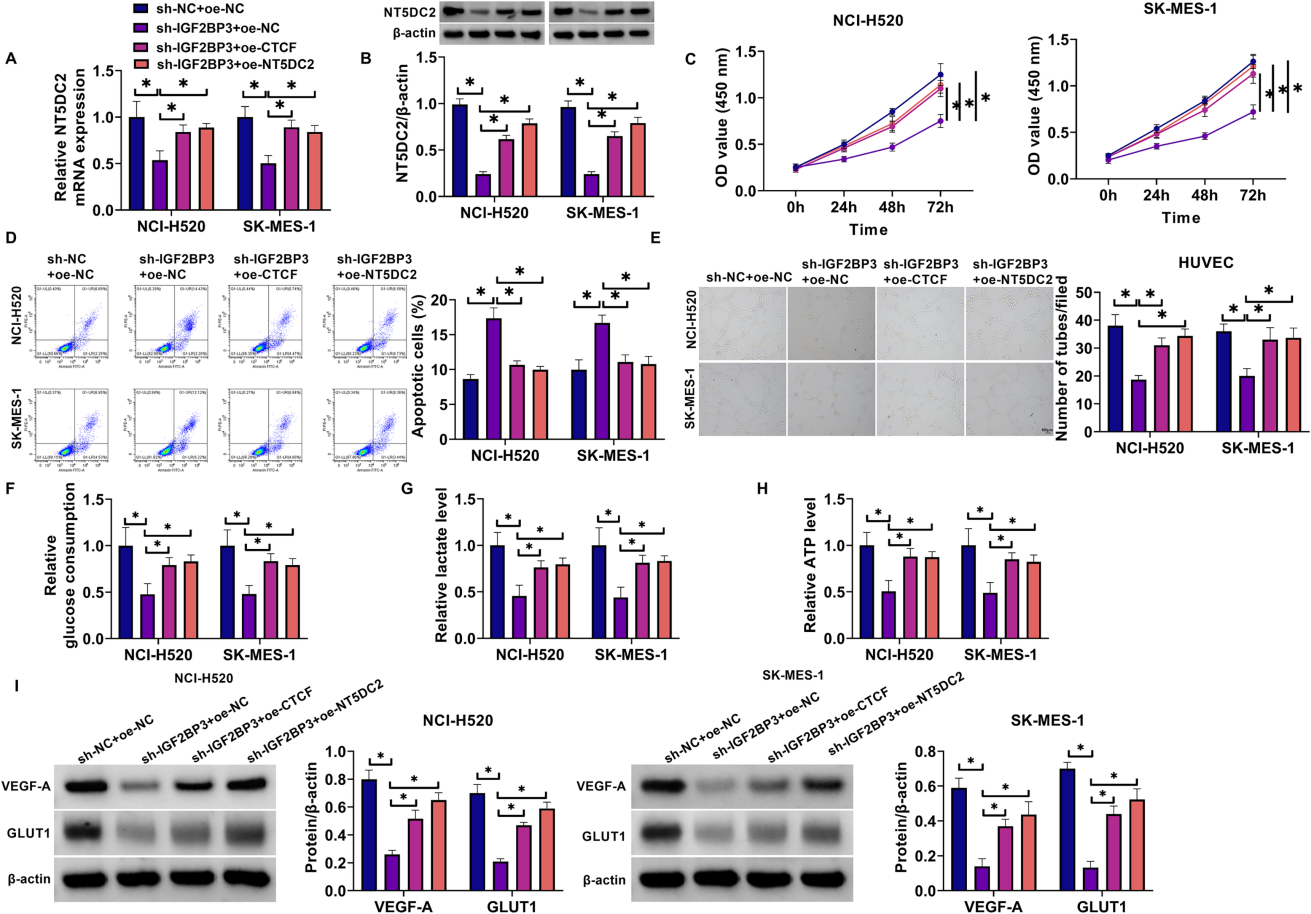
Overexpression of CTCF or NT5DC2 attenuated IGF2BP3 silencing–induced effects on LUSC cell proliferation, tube formation, glycolysis, tumor formation and cell apoptosis. Both NCI‐520 and SK‐MES‐1 cells were divided into sh‐NC + oe‐NC group, sh‐IGF2BP3 + oe‐NC group, sh‐IGF2BP3 + oe‐CTCF group, and sh‐IGF2BP3 + oe‐NT5DC2 group. (A) NT5DC2 mRNA expression was analyzed by qRT‐PCR. (B) NT5DC2 protein expression was assessed by western blotting assay. (C) CCK‐8 assay was conducted to determine cell proliferation. (D) Flow cytometry was applied to analyze cell apoptosis. (E) Tube formation was analyzed through the tube formation assay. (F–H) Colorimetric methods were used to analyze glucose consumption, lactate production and ATP levels. (I) VEGF‐A and GLUT1 protein expression were analyzed through the western blotting assay. *P < 0.05.

**FIGURE 7:**
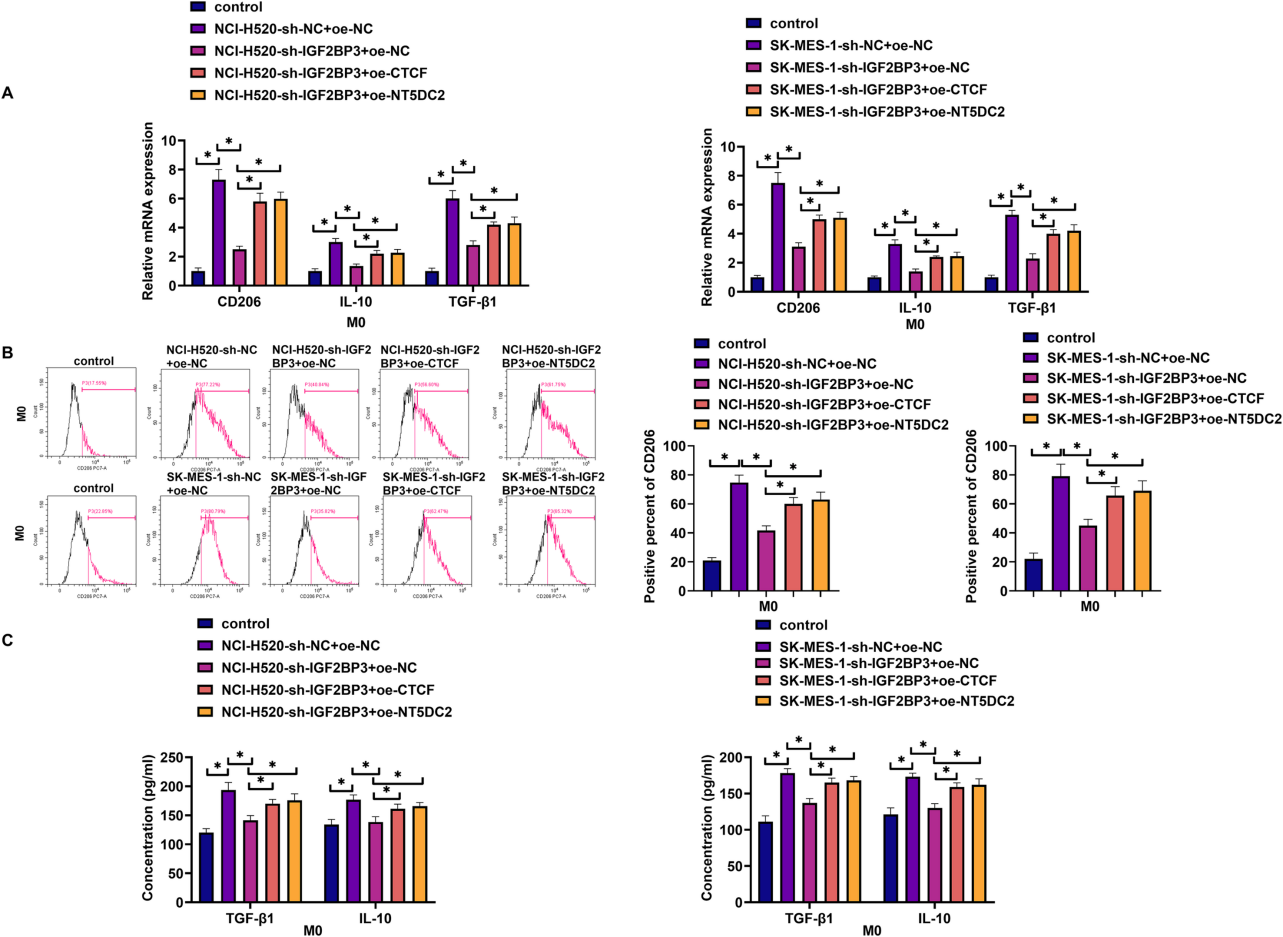
Overexpression of CTCF or NT5DC2 attenuated IGF2BP3 silencing–induced effects on M2 macrophage polarization. THP‐1 cells were cultured in the bottom layer of transwell plates and induced with PMA for 24 h to differentiate into macrophages. After washing with PBS to remove PMA, the NCI‐520 and SK‐MES‐1 cells transfected with sh‐NT5DC2, sh‐NC, oe‐CTCF, oe‐NT5DC2, or oe‐NC were inoculated into the upper layer of the transwell plates and co‐cultured with THP‐1 cells induced by PMA for 48 h. (A) The mRNA expression of CD206, IL‐10 and TGF‐β1 was analyzed by qRT‐PCR. (B) Flow cytometry was applied to detect CD206 expression. (C) ELISAs were performed to detect the levels of TGF‐β1 and IL‐10. *P < 0.05.

The study further analyzed whether CTCF or NT5DC2 was involved in the regulation of IGF2BP3‐induced effects on the malignant progression of LUSC cells. To achieve this, the study downregulated IGF2BP3 expression and upregulated both CTCF and NT5DC2 expression in both NCI‐520 and SK‐MES‐1 cells. The results first showed that IGF2BP3 silencing downregulated NT5DC2 expression at mRNA and protein levels, whereas the effects were relieved after transfection with overexpression plasmid of CTCF or NT5DC2 (Figure 6A,B). Subsequently, IGF2BP3 knockdown inhibited cell proliferation and induced cell apoptosis, whereas these effects were attenuated after transfection with overexpression plasmid of CTCF or NT5DC2 (Figure 6C–D). IGF2BP3 silencing inhibited tube formation and decreased glucose consumption, lactate production, and ATP levels (Figure 6E–H). IGF2BP3 silencing also led to decreases in VEGF‐A and GLUT1 levels (Figure 6I). However, these effects were counteracted after CTCF or NT5DC2 overexpression (Figure 6E–I). Further, the co‐culture of IGF2BP3‐deficient cells with M0 cells led to decreased levels of CD206, IL‐10 and TGF‐β1. In contrast, when these deficient cells were co‐cultured with CTCF or NT5DC2‐overexpressing cells, the opposite effects were observed (Figure 7A–C). Thus, overexpression of CTCF or NT5DC2 counteracts IGF2BP3 silencing–induced effects in LUSC cells.

## 4 Discussion

According to Gene Ontology annotations, NT5DC2 shows a notable capacity to bind to metal ions and exhibits intrinsic 5′‐nucleotidase enzymatic activity. Previous data have revealed that NT5DC2 can bind to Fyn and thus promote cancer progression [30]. It also participates in cancer progression by regulating the ECM‐receptor interaction pathway [31]. In addition, NT5DC2 mediates the malignant growth of breast cancer by blocking the EGFR pathway [18]. However, its role and mechanism in the progression of lung cancer, especially LUSC, remains unclear. The present work was performed to analyze its specific role in the malignant phenotypes of LUSC cells and the underlying mechanism. Herein, the results showed that NT5DC2 contributed to the malignant progression of LUSC, and the detailed mechanism involved the regulation of the IGF2BP3/CTCF axis in its expression.

Previous data have revealed the promoting effects of NT5DC2 on NSCLC cell proliferation, migration, and invasion [17]. The work was the first one to analyze NT5DC2's role in LUSC. The result showed a high expression of NT5DC2 in LUSC tissues and cells. In addition, NT5DC2 was a valuable biological marker in predicting the prognosis of LUSC. NT5DC2 silencing inhibited LUSC cell proliferation, tube formation, and glycolysis and induced cell apoptosis in vitro. Moreover, its depletion delayed tumor formation in vivo. NT5DC2 knockdown inhibited M2 macrophage polarization. p53 acts as a tumor suppressor by regulating cell apoptosis, cell differentiation, cell invasiveness, cell cycle, and TEM signaling [32]. As reported in a paper, p53 activation inhibits M2‐type macrophage polarization in myeloid lineage [33]. A previous study has revealed the regulation of NT5DC2 in the p53 signaling was responsible for its promoting effect on NSCLC progression [17]. Thus, NT5DC2 may inactivate the p53 signaling to induce M2 macrophage polarization, thus promoting the malignant progression of LUSC.

In terms of mechanism, IGF2BP3 regulates multiple genes by binding to m6A‐modified RNA and affects various cellular processes. For example, IGF2BP3 stabilized TWIST1 expression in an m6A‐dependent manner and thus promoted the proliferation, migration, and invasion of NSCLC cells [34]. IGF2BP3 stabilized TMBIM6 mRNA through the m6A modification and thus promoted the growth and metastasis of NSCLC cells [35]. The present work indicated that IGF2BP3 was upregulated in LUSC tissues and cells. IGF2BP3 silencing inhibited LUSC cell proliferation, tube formation, glucose metabolism and M2 macrophage polarization and induced cell apoptosis. Moreover, IGF2BP3 upregulated CTCF mRNA expression, and the detailed mechanism involved its regulation in CTCF mRNA stability through the m6A modification.

Genetic alterations in CTCF have been observed in numerous cancers, including colorectal cancer [36] and breast cancer [37]. In lung cancer progression, it has been found that CTCF contributed to the proliferation and metastasis of lung cancer cells by binding to the promoter region of prominin 2 [27]. We identified CTCF as a transcriptional regulator of NT5DC2 in LUSC cells. In addition, CTCF was upregulated in lung cancer tissues and cells. Moreover, CTCF overexpression promoted LUSC cell proliferation, tube formation, glucose metabolism and M2 macrophage polarization and inhibited cell apoptosis. Overexpression of NT5DC2 or CTCF attenuated IGF2BP3 silencing–induced effects in LUSC cells. Thus, the CTCF/NT5DC2 axis was essential for the regulation of IGF2BP3 during the malignant progression of LUSC cells.

However, the study may be limited by the size of the clinical sample population used to assess NT5DC2 expression in LUSC tissues. A larger sample size could provide more robust statistical evidence for the correlation between NT5DC2 expression and disease progression. In addition, the study employed various functional assays to analyze the influence of NT5DC2 on tube formation, glycolysis, M2 macrophage polarization, and cell proliferation. However, these assays represent a controlled laboratory environment and may not fully reflect the complexity of tumor behavior in a dynamic physiological context.

Thus, IGF2BP3 increased CTCF expression to promote the transcriptional process of NT5DC2, thus accelerating M2 macrophage polarization. This enhancement of NT5DC2 expression promoted cell proliferation, tube formation and glucose metabolism and inhibited cell apoptosis, ultimately promoting the malignant progression of LUSC (Figure S1). Understanding the role of IGF2BP3, CTCF, and NT5DC2 in the pathogenesis of LUSC opens up new avenues for therapeutic intervention. Targeting this signaling axis could potentially lead to the development of novel treatments that slow or reverse the malignant progression of LUSC.

## Author Contributions

Jifeng Sun conducted the experiments and drafted the manuscript. Hao Wang and Ran Zhang collected and analyzed the data. Xiaoxuan Sun contributed the methodology, operated the software and edited the manuscript. Jun Wang and Yuwen Wang designed and supervised the study. All authors reviewed the manuscript.

## Conflicts of Interest

The authors declare no conflicts of interest.

## Supporting information


**Figure S1** **The mechanism of IGF2BP2 regulating the malignant progression of LUSC.** IGF2BP3 increased CTCF expression to promote the transcriptional process of NT5DC2, thus accelerating M2 macrophage polarization, increasing cell proliferation, tube formation and glucose metabolism and inhibiting cell apoptosis, ultimately promoting the malignant progression of LUSC.

**Table S1** Clinical characteristics of patients with LUSC.

## Data Availability

The data are available from the corresponding author on reasonable request.
