# Supplementary figures and images for: IGF2BP3/CTCF Axis–Dependent NT5DC2 Promotes M2 Macrophage Polarization to Enhance the Malignant Progression of Lung Squamous Cell Carcinomas
Source: Clin Respir J. 2024 Nov 6;18(11):e70031. doi: 10.1111/crj.70031 (PMC11540834; doi:10.1111/crj.70031)

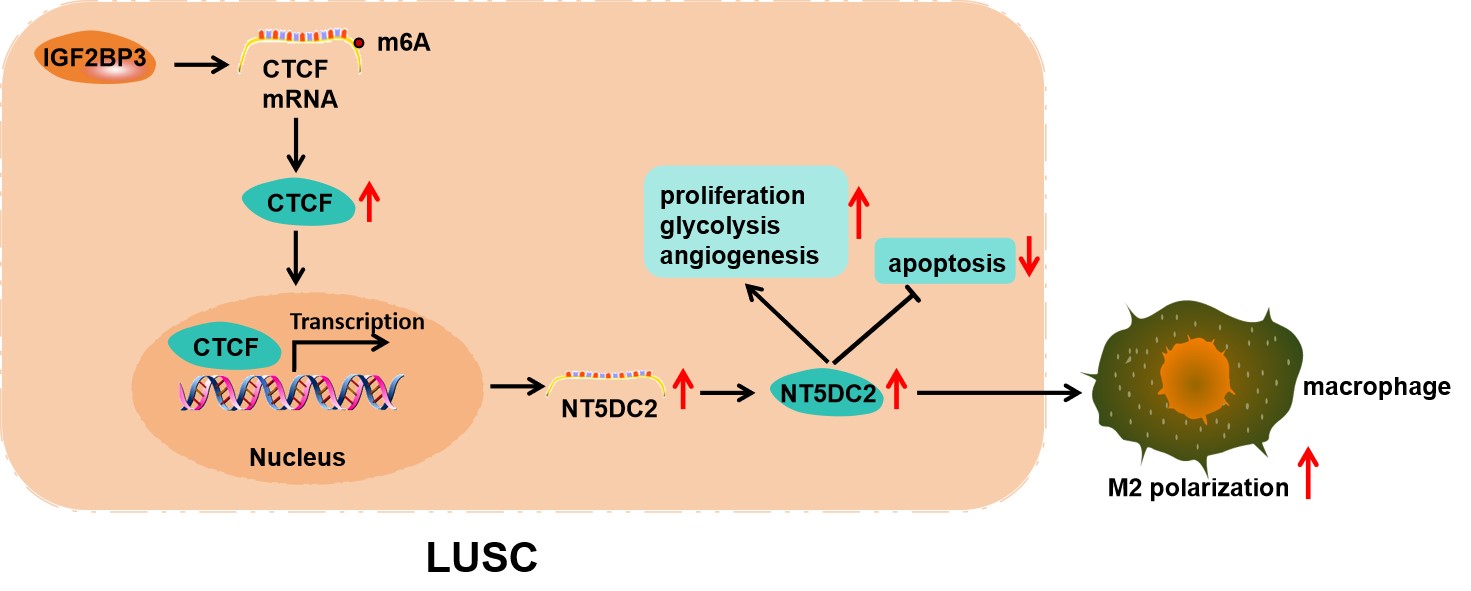

Supplement: Supplementary file 1 — Figure S1 The mechanism of IGF2BP2 regulating the malignant progression of LUSC. IGF2BP3 increased CTCF expression to promote the transcriptional process of NT5DC2, thus accelerating M2 macrophage polarization, increasing cell proliferation, tube formation and glucose metabolism and inhibiting cell apoptosis, ultimately promoting the malignant progression of LUSC. [file CRJ-18-e70031-s002.jpg]
