# Supplementary material for: IGF2BP3/CTCF Axis–Dependent NT5DC2 Promotes M2 Macrophage Polarization to Enhance the Malignant Progression of Lung Squamous Cell Carcinomas
Source: Clin Respir J. 2024 Nov 6;18(11):e70031. doi: 10.1111/crj.70031 (PMC11540834; doi:10.1111/crj.70031)
Supplement: Supplementary file 2 — Table S1 Clinical characteristics of patients with LUSC. [file CRJ-18-e70031-s001.docx]

**Table S1 Clinical characteristics of patients with LUSC**

| Variable | Case (%) |
| --- | --- |
| Age  ≤60 years  ＞60 years | 12 (33.33%)  24 (66.67%) |
| Sex  Male  female | 31 (86.11%)  5 (13.89%) |
| Smoking  Smoker  No Smoking  Clinical stage  І-ІІ | 25 (69.44%)  11 (30.56%）  21 (58.33%) |
| ІІІ-ІV | 15 (41.67%) |
| Lymph node metastasis |  |
| N0 | 23 (63.89%) |
| N1-N3 | 13 (36.11%) |
